# The role of epigenetics in respiratory health in urban populations in low and middle-income countries

**DOI:** 10.1017/gheg.2019.7

**Published:** 2019-11-26

**Authors:** Nicole M. Robertson, Alex Kayongo, Trishul Siddharthan, Suzanne L. Pollard, Jose Gomez Villalobos, Christine Ladd-Acosta, Bruce Kirenga, William Checkley

**Affiliations:** 1 Division of Pulmonary and Critical Care Medicine, Johns Hopkins University, Baltimore,MD, USA; 2 Center for Global Non-Communicable Disease Research and Training, Johns Hopkins University, Baltimore,MD, USA; 3 Department of Immunology and Molecular Biology, Makerere University College of Health Sciences, Kampala, Uganda; 4 Division of Pulmonary and Critical Care, Yale University, New Haven,CT, USA; 5 Department of Epidemiology, Bloomberg School of Public Health, Johns Hopkins University, Baltimore,MD, USA; 6 Division of Pulmonary Medicine, Makerere University Lung Institute, Mulago Hospital, Makerere University, Kampala, Uganda

**Keywords:** Chronic respiratory diseases, epigenetics, low and middle-income countries, slum dwellers

## Abstract

As urbanization increases in low- and middle-income countries (LMICs), urban populations will be increasingly exposed to a range of environmental risk factors for non-communicable diseases. Inadequate living conditions in urban settings may influence mechanisms that regulate gene expression, leading to the development of non-communicable respiratory diseases. We conducted a systematic review of the literature to assess the relationship between respiratory health and epigenetic factors to urban environmental exposures observed in LMICs using MEDLINE, PubMed, EMBASE, and Google Scholar searching a combination of the terms: epigenetics, chronic respiratory diseases (CRDs), lung development, chronic obstructive airway disease, and asthma. A total of 2835 articles were obtained, and 48 articles were included in this review. We found that environmental factors during early development are related to epigenetic effects that may be associated with a higher risk of CRDs. Epigenetic dysregulation of gene expression of the histone deacetylase (HDAC) and histone acetyltransferase gene families was likely involved in lung health of slum dwellers. Respiratory-related environmental exposures influence HDAC function and deoxyribonucleic acid methylation and are important risk factors in the development of CRD. Additional epigenetic research is needed to improve our understanding of associations between environmental exposures and non-communicable respiratory diseases.

## Introduction

In 2014, 881 million people in low- and middle-income countries (LMICs) lived in urban slums, and this number is expected to grow on average by 9 million people each year [1, 2]. A greater proliferation of low-income settlements, also known as slums, exists with a lack of access to sustainable housing, expansive living space, sanitation, safe drinking water, and security [3, 4]. Disparities are present among slum dwellers but they are not equally distributed worldwide. Specifically, in sub-Saharan Africa, the United Nations Habitat estimated that a majority of urban populations, at about 56%, live in slums [2, 5]. Overall, Africa is shifting to a predominantly urban continent. It is estimated that 40% of Africa's population lives in urban areas, but by 2030 one out of two individuals living in sub-Saharan Africa will live in an urban area [6]. Urbanization, the migration of residents from rural to urban areas often for the economic opportunities offered by urban development, has further contributed to the increase in urban poverty in LMICs [3]. This rapid urbanization has been accompanied by significant shifts in health patterns, increasing the prevalence of non-communicable diseases (NCDs). Lung disease is known as a leading cause of mortality in LMICs, where it is reported to account for 15% of all deaths [7].

As the prevalence of asthma and chronic obstructive airway disease (COPD) rise, the WHO predicts that deaths due to NCDs will increase by 27% by 2030 [7–10]. This is mainly due to demographic transitions and changing lifestyles of populations associated with urbanization. Moreover, populations in urban slums often have inadequate access to health services and seek care after symptoms have advanced and often when life-threatening complications have arisen [11]. The combination of slum conditions in Bangladesh, Kenya, and Egypt has led to severe malnutrition in adolescents, primed by a lower under-five child nutrition status, when compared to individuals living in rural settings [8, 12, 13]. As a result, slum living has been linked to poverty and multiple risk factors for respiratory disease including poor housing quality, overcrowding, traffic-related and household air pollution, tobacco smoke, psychological stress, occupational exposures, and exposure to allergens and malnutrition [4, 7, 11, 12].

Environmental exposures associated with living in slums can lead to epigenetic modifications and changes in cell function that begin *in utero* and could last a lifetime, therefore predisposing slum residents to poor respiratory health outcomes. Epigenetics is the study of mitotically-heritable phenotypes not resulting from changes in the deoxyribonucleic acid (DNA) sequence. The term epi- is a Greek suffix meaning ‘on top or outside’, which highlights the nature of the mechanisms outside of the DNA code [14]. The most widely known epigenetic marks include: DNA methylation, histone tail modifications, and noncoding ribonucleic acids (RNAs) [15–17]. These mechanisms control access to DNA to participate in transcription [15, 17]. The same principle applies to histone tail regulatory mechanisms as acetylation by histone acetyltransferases (HATs) leading to active gene transcription, whereas deacetylation by histone deacetylases (HDACs) leads to gene silencing [18, 19]. Non-coding RNAs (ncRNAs) also play a critical role in regulating gene expression by controlling gene expression through binding of 3′ untranslated regions of mRNA [20]. The end result is either mRNA degradation or inhibition of protein translation.

Epigenetic markers are important regulators of overall DNA stability, cell differentiation, imprinting, and organismal development. As a result, epigenetic mechanisms, through their key role in cell function and heritable nature, have been implicated in the development of chronic respiratory diseases (CRDs) that include asthma and COPD in people living in LMICs, predisposing these populations to significant negative health outcomes [15]. However, potential molecular mechanisms due to epigenetic modifications from environmental exposure in this population are currently lacking. In this systematic review, we aim to focus on the current knowledge of epigenetic markers related to human environmental exposures that affect lung health and relate this to urban residents, but more specifically slum dwellers, in LMICs as a measure of the impact of urbanization in chronic illness. Also, we aim to identify the knowledge gaps in interactions between epigenetic mechanisms and specific threats to respiratory health in LMIC settings.

## Methods

**Fig. 1.**
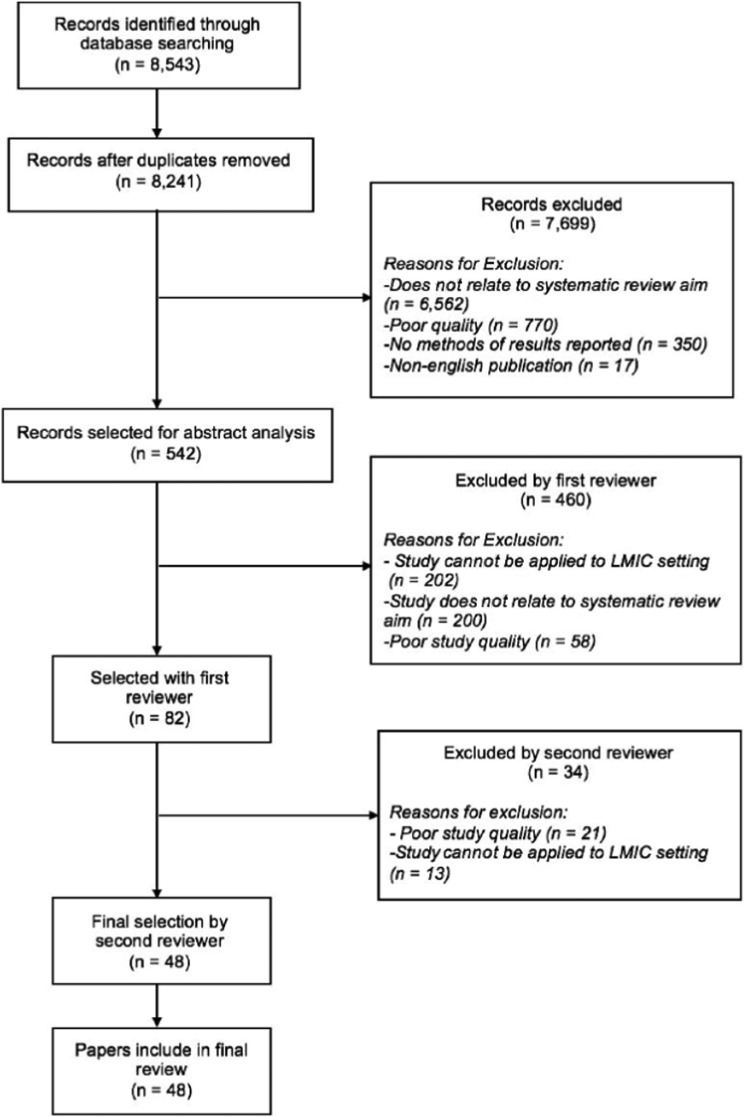
Literature search process to identify papers to include in this review.

Using MEDLINE, PubMed, EMBASE, and Google Scholar and following the Preferred Reporting Items for Systematic Reviews and Meta-Analyses guidelines [21], we conducted a systematic search of literature published between 1 January 1995 and 9 September 2017 using the following search terms: epigenetics, Africa, Asia, Latin America, Caribbean, LMICs, CRDs, NCDs, urban slum, lung development, and asthma using a combination of ‘AND’ and ‘OR’ search settings among various combinations of terms. The primary outcomes of the review are epigenetic markers and mechanisms related to the following human environmental exposures that influence lung health: cigarette smoke, second hand smoke, ambient or household air pollution, traffic-related pollution, maternal nutrition, exposure to allergens, and nutritional status. The generated abstracts were limited to publications in English and to research papers, editorials, reviews, original articles, and reports. The first reviewer (NR) screened titles and abstracts for relevance, selecting papers for abstract analysis. The inclusion and exclusion criteria were considered during this process. Only research articles relating to settings in LMICs or studies that could be applied to populations in LMICs were included. Articles published before 1995 were excluded from our search to limit our review to recent literature. The first reviewer (NR) selected 82 papers. Reference lists of selected papers were manually searched for further related studies. Then, the second reviewer (AK) performed the final selection of 48 articles. Any disagreement between the two authors resulted in a discussion and joint review of the article with reconciliation. Quality criteria were assessed using the Newcastle-Ottawa Scale (NOS) [22]. The aim of the search was to assess the relationship between respiratory health and epigenetic factors in environments similar to that of urban areas of LMICs. The methodological summary of this literature search is outlined in Fig. 1.

## Results

A total of 8543 articles were obtained from the searches, with 48 articles ultimately included in this review. Abstracts were manually sorted, and unrelated and duplicated papers were removed. Papers were excluded based on quality, relevance to systematic literature review aims, and reported methodology and results (Fig. 1). Studies that presented results not conducted in LMICs or that could not be applied to LMIC settings were excluded. Papers that did not report methodology or results were excluded. Other reasons that papers were excluded were duplication or abstracts and publications that were not written in English. The quality of papers was assessed utilizing the NOS, Table 1. The articles included in this review span a diverse geographical distribution and are included in Supplementary Table S1 with specific relationships between epigenetic factors and lung health further outlined in Table 2. Our systematic literature review yielded articles relating to epigenetic modifications due to environmental exposures including cigarette smoke, air pollution, maternal nutrition, exposure to allergens, and malnutrition, among others. Few studies related specifically to how these predisposing environmental factors were associated with common CRDs including COPD and asthma.

**Table 1.** Example of NOS for assessment of quality of included studies-cross-sectional studies (1 indicates individual criterion within the subsection was fulfilled)

Quality assessment criteria	White *et al*. [23]	Waterland *et al*. [24]	Ezzie *et al*. [25]	Kohli *et al*. [26]
Selection (maximum 4)				
Case definition adequate	1	1	1	1
Representativeness of the cases	1	1	1	1
Selection of controls	1	1	1	1
Definition of controls	1	1	0	1
Comparability (maximum 2)				
Comparability of cohorts on the basis of the design of analysis (controls used)	1	1	1	1
Confounding factors are controlled.	1	1	1	1
Exposure (maximum 3)				
Ascertainment of exposure	1	1	1	1
Same method cases and controls?	1	1	1	1
Non-response rate	0	1	0	0
Overall quality score (maximum 9)	8	9	7	8

**Table 2.** Results of the relationship between epigenetic factors and lung health in included studies in this systematic literature review

Epigenetic factors	Activity in asthma, COPD, or smoking	Reference	Sample size	Type of sample	Country
Class 1 HDAC Family					
HDAC 1	Decreased in asthma, not yet studied in smoking and COPD patients	[27]	40	Bronchial biopsy samples	UK
		[28]	28	Alveolar macrophages	UK
		[29]	24	Rat lung tissue	UK
HDAC 2	Decreased in asthma, smoking and COPD	[27]	40	Bronchial biopsy samples	UK
		[28]	28	Alveolar macrophages	UK
		[29]	24	Rat lung tissue	UK
		[30]	29	Human bronchial biopsies	UK
HDAC 3 and 8	Decreased in COPD, no difference in asthma, limited published data in smoking	[28]	28	Alveolar macrophages	UK
		[31]	159	Human lung tissue	USA and Canada
Class II HDAC family					
HDAC 5	Decreased in COPD, no difference in asthma, limited published data in smoking	[28]	28	Alveolar macrophages	UK
		[31]	159	Human lung tissue	USA and Canada
Class III HDAC family					
SIRT1	Limited published data				
Class IV HDAC family					
HDAC 11	Limited published data				
GNATs-HAT family					
HAT1, GCN5, GCN5L, Elp3, PCAF	Increased in asthma patients, limited published data on smoking and COPD	[27]	40	Bronchial biopsy samples	UK
		[32]	–	Cell culture-mature monocytes	USA
General transcription factor-HATs					
TAF250, TFIIIC	Increased in asthma, limited published data on smoking and COPD	[27]	40	Bronchial biopsy samples	UK
		[28]	28	Alveolar macrophages	UK

HDAC, histone deacetylase; SIRT1, sirtuin; HAT, histone acetyl transferase; GCN5, general control of amino acid synthesis protein 5; GCN5L, general control of amino acid synthesis protein 5 ligand; Elp3, elongation complex protein; PCAF, protein300/CREB-binding protein-associated factor; TAF250, TBP-associated factor 250 kDa; TFIIIC, transcription factors IIIC.

### DNA methylation and non-coding RNAs

Our search revealed several slum-related exposures *in utero*, in childhood, and adulthood, which have been associated with altered DNA methylation. We found that cigarette smoke and second-hand smoke (SHS) are both associated with altered epigenome methylation patterns. *In utero* exposure to maternal tobacco smoke has also been shown to result in both global and site-specific DNA methylation levels during fetal development [33–36]. Exposure to SHS has shown increased methylation of CpG sites in T effector cell IFN-*γ* promoters relative to unexposed individuals [26]. Traffic-related air pollution exposure increased locus-specific methylation in the *TET1* promoter region, specifically at CpG (cg23602092) site, which was significantly associated with childhood asthma [37]. Studies *in utero* have shown that maternal famine was associated with higher methylation of *IL10*, *LEP*, *ABCA1*, *GNASAS*, *and MEG3* genes, compared to the same-sex unexposed siblings, potentially increasing risk of asthma later in life [38–40]. In the current literature, there is a gap regarding ncRNA's role in regulating gene expression emphasizing the need for greater research in this area.

### Histone deacetylases and histone acetyltransferases

Two major enzymes, HDAC and HATs, potentially play a role in epigenetic factors related to the lung health of slum dwellers. The HDAC family facilitates local histone deacetylation and transcriptional repression by binding to DNA substrates. In Class I of the HDAC family, specifically HDAC 1, 2, 3, and 8, have been shown at reduced levels in individuals with COPD or asthma or individuals that smoke tobacco. Marwick *et al*., observed decreased HDAC2 activity due to cigarette smoke exposure. Ito *et al*., expanded on these results by demonstrating that cigarette smoke exposure decreased overall HDAC activity in bronchial biopsies and alveolar macrophages of smokers compared to non-smokers [29, 30]. Moreover, the HAT families acetylate the lysine residues at the N-terminus of histone proteins by removing positive charges. As a result, the affinity between histones and DNA is reduced, facilitating access to the promoter region of target genes by RNA polymerase and transcription factors. Among the Gcn5-related N-acetyltransferase (GNAT) family, HAT activity was associated with higher pro-inflammatory gene expression among individuals with asthma [27]. A summary of these findings included studies is contained in Table 2 and in Supplementary Table S1.

## Discussion

In this review, maternal nutrition, tobacco smoking, ambient and household air pollution, and traffic-related air pollution during early development were risk factors associated with epigenetic changes in LMICs. In particular, the HDAC, HAT, and GNAT families, specifically, were associated with lung health outcomes.

The Barker hypothesis proposes that environmental factors acting during the phase of early development interact with the genome to change the capacity of the organism to cope with its environment in later life [38, 41]. This hypothesis has expanded to find other immunological, mental health, and reproductive diseases that can result from malnutrition or over-nutrition [38]. Current research points to evidence that epigenetic marks alter gene expression in the lung, which may be associated with common CRDs [42]. Because the epigenome is dynamic and changes accordingly to the environment and ageing process, associations between cigarette smoke and SHS and epigenetic marks have been demonstrated in recent studies in high-income settings [33, 43]. For example, intergeneration DNA methylation associated with *in utero* cigarette smoke exposure is associated with a high risk of asthma development in offspring [33, 44]. *In utero* exposure to maternal tobacco smoking has been shown to be significantly associated with increased newborn epigenome-wide alterations and methylation at 26 CpGs mapped at 10 genes and reduced mean methylation for AluYb8 repetitive elements [33–35]. SHS exposure has also been associated with a higher percentage of methylation of CpG sites in the T effector cell IFN-*γ* promoter with a concomitant reduction in IFN-*γ* expression from T effector cells when compared to individuals not exposed to SHS [26]. Exposure to cigarette smoke also affects miRNA expression. Ezzie *et al*., found that *miR-223* and *miR-1274a* were expressed almost three-fold in COPD lung samples compared to non-COPD lung samples [25]. Moreover, 28 miRNAs were differentially expressed in airway epithelial cells, with most of these miRNAs down-regulated when exposed to tobacco smoke compared to unexposed cells [45]. Because childhood years have been found to be a critical time for rapid lung development, reducing exposure to cigarette smoke and SHS may reduce the risk of developing chronic respiratory conditions later in life [46].

Air pollution is a ubiquitous environmental exposure, especially in urban settings. Previous research in high-income settings has found that DNA methylation of long interspersed nucleotide element (LINE)-1 was lower with higher levels of ambient particulate matter [47]. Diesel exhaust particle exposure has been found to impact DNA methylation at 2827 CpG sites compared to filtered air; with CpG sites, such as *GSTP1*, becoming significantly less methylated [48]. Asthma was significantly associated with loss of methylation at a single CpG site in the *TET1* promoter (cg23602092) and increased global 5hmC in bronchial epithelial cells and human participants [37]. Diesel exhaust particle exposure was associated with alterations in Alu and LINE-1 elements and the CpG site within *miR-21*, and increased FOXP3 was significantly associated with increased diesel exhaust particle exposure, which was associated with increased risk of asthma development in children [48, 49]. Allergen exposure, diesel exhaust particle exposure, and co-exposure showed alterations in 7 CpG sites in bronchial epithelial cells after two days. Spacing out allergen and diesel exhaust particle exposure by four weeks resulted in alterations in over 500 CpG sites including four Hox family of genes such as *HOXA3*, *HOXA4*, *HOXB1*, and *HOXB3* involved in fetal lung development, and global DNA methylation suggesting sequential exposure has a pronounced impact on DNA methylation [50]. Moreover, methylation of the *ACSL3* 5′-CGI was significantly associated with maternal airborne polycyclic aromatic hydrocarbon exposure above 2.41 ng/m^3^ in umbilical cord white blood cells potentially related to the development of traffic-related air pollution exposure asthma [51].

Diet and nutrition may also exert epigenetic changes. Indeed, the relationship between epigenetic maternal and either early childhood nutrition or dietary supplementation have been studied in populations in the Dutch Hunger Winter Famine in Europe, in Nepal, and in The Gambia [24, 40, 52, 53]. In individuals exposed to *in utero* famine conditions *INSIGF* methylation was significantly reduced and increased in *IL10*, *LEP*, *ABCA1*, *GNASAS*, *and MEG3* genes compared to unexposed same-sex siblings [40]. Seasonal changes in maternal methyl-donor nutrient intake during conception also have been found to influence 13 plasma biomarkers and systemic epigenetic changes in human metastable epialleles in Gambian populations with metastable epialleles having increased DNA methylation levels for individuals conceived in the rainy season when nutritional intake is low [24, 52]. Moreover, the lack of maternal dietary vitamin A may alter *in utero* lung development potentially increasing susceptibility of postnatal lung diseases. In Nepal, children aged 9–13 years born to mothers who were assigned to receive 7000-μg retinol-equivalents of vitamin A weekly before, during, and after pregnancy were shown to have a larger forced expiratory volume at 1 s and forced vital capacity than children of the same age who were born to mothers assigned to receive a placebo [53].

Emerging evidence of the relationship between environmental exposures and epigenetic markers coupled with the function of epigenetic regulation in T-cell differentiation demonstrates epigenetic alterations that may contribute to a higher prevalence of asthma in LMICs [54]. Current research is focusing on the effect of maternal nutrition on the development of atopy and asthma in children [55, 56]. For example, epigenetic changes, specifically increasing site-specific methylation levels of the genome, during pregnancy shifts towards a Type II helper phenotype, increasing the risk of asthma [38, 39]. Previous epidemiological studies have shown that increasing folate levels, which is a precursor for the methyl group supply that is used to generate DNA methylation marks, are associated with an increased risk of developing asthma [38]. Additionally, acetylation of histones may influence the onset of asthma because increased levels of acetylation of H4 has been found in asthmatic individuals and is associated with increased inflammatory gene expression in lung tissue [57]. The level of acetylation of histones has been shown to be associated with enhanced inflammatory gene expression by HATs and reduced inflammatory gene expression by HDACs [58]. The altered HAT/HDAC ratio from inflammation in peripheral blood cells has been shown in adults and children that correlates with alterations in bronchial hyperresponsiveness as in asthma and in patients with COPD [59, 60].

The degree of increase in the acetylation of histones associated with the promoter region of inflammatory genes in peripheral lung tissue has been found to be associated with the severity of COPD [61]. Alterations in histone acetylation patterns and other epigenetic changes could mean promising therapies for anti-inflammatory conditions such as corticosteroid resistant cases of asthma [57]. Glucocorticoids change acetylation patterns of histones via mechanisms that regulate inflammatory and anti-inflammatory genes [57]. In addition to maternal nutrition, cigarette smoke exposure, for example, reduces the expression of HDAC2, a glucocorticoid receptor corepressor, at the protein and mRNA levels meaning maternal secondhand smoke exposure may influence the HDAC2 promoter, which could lead to asthma progression *in utero* [62].

While there is a significant amount of research focused on the genetics and epigenetic factors surrounding populations in high-income countries, there is little priority on the interaction between environmental factors and genetics in LMICs, where the burden of disease is greatest. The lack of research data, as evident in a large number of papers excluded during the systematic review process, hinders health systems from allocating resources and targeting policy efforts towards short-term and long-term CRD prevention and management services in LMICs (Fig. 1). Further epigenetic research could lead to identifying epigenetic targets stemming from living in urban slums. This research could identify potential therapies that target epigenetic modifications in urban slum dwellers along with associated poor health outcomes that result from epigenetic modifications.

Currently, there is little focus on these silent killers known as NCDs such as CRDs in LMICs. As is evident in this review, environmental factors afflicting residents of slums and urban areas of developing settings can have a significant impact on lung health and development that can lead to adverse health outcomes. Environmental exposures *in utero* and during adolescent years can have long lasting and possibly irreversible effects later in life. Researching and targeting the upstream factors of CRD onset and the biological mechanisms is not only economical but will improve the quality of life for these vulnerable, at-risk populations.

## Supplementary material

For supplementary material accompanying this paper visit http://dx.doi.org/10.1017/gheg.2019.7. click here to view supplementary material
