# Supplementary material for: The role of epigenetics in respiratory health in urban populations in low and middle-income countries
Source: Glob Health Epidemiol Genom. 2019 Nov 26;4:e8. doi: 10.1017/gheg.2019.7 (PMC6983949; doi:10.1017/gheg.2019.7)
Supplement: Supplementary file 1 [file S2054420019000071sup001.pdf]

Supplementary Information: Summary of Systematic Review Included Studies

Study Details	Study Design/ Sample	Exposure(s)	Measurement	Main Finding(s)	Possible Links Assessed with CRDs
Ito et al., 2001	Biopsy samples and bronchoalveolar lavage macrophages of 16 non-smokers and 13 healthy smokers, which were age-matched<br><br>United Kingdom	Cigarette smoke	HDAC activity; TNF-α release; expression of inflammatory mediators	Cigarette smoke decreases the expression of HDAC2 and HDAC activity in biopsies and alveolar macrophages. Cigarette smoke increased IL-1β–induced expression of TNF-α in alveolar macrophages with TNF-α release increased correlating with HDAC activity.	Cigarette smoke may reduce HDAC expression potentially leading to increased expression of inflammatory mediators explaining differences between smokers and non-smokers with COPD.
Ito et al., 2002 *American Journal of Respiratory and Critical Care Medicine*	HDAC and HAT activity and expression in bronchial biopsies from healthy and asthmatic participants<br><br>United Kingdom	Inhaled corticosteroids	HDAC and HAT activity and expression levels	Individuals with asthma had decreased HDAC activity (decreased expression of HDAC1 and HDAC2) in bronchial biopsy relative to healthy controls. Asthmatics taking corticosteroids had increased HDAC and decreased HAT activity in in biopsy samples.	Increased HAT activity and decreased HDAC activity in asthmatics may be due to increased expression of multiple inflammatory genes.
Ito et al., 2002 *Proceedings of the National Academy of Sciences of the USA*	Double-blind crossover controlled study to evaluate the effect of low-dose theophylline on HDAC activity<br><br>United Kingdom	Low-dose theophylline	HDAC levels; fiberoptic bronchoscopy; collection of bronchoalveolar lavage macrophages	Low-dose theophylline acts by increasing activation of HDACs to suppress inflammatory genes.	COPD and asthma medications may enhance HDAC activity in epithelial cells and macrophages.
Cosío et al., 2004	Alevolar macrophages and peripheral blood mononuclear cells in 28 participants consisting of 10, 10, 8, and 10 participants with intermittent asthma, persistent asthma, and	Asthma (persistent or intermittent) or no asthma (healthy control)	Levels of HDACs and HATs in alveolar macrophages and peripheral blood mononuclear cells	HDAC activity was reduced in individuals with asthma compared to controls.	Alveolar macrophages from asthmatics showed reduced HDAC and elevated HAT activity, which is associated with increased transcription of inflammatory proteins

	controls respectively<br><br>United Kingdom				leading to the inflammatory cascade in asthmatic airways
Hogg et al., 2004	Lung tissue samples of 159 patients consisting of 39 with stage 0, 39 with stage 1, 22 with stage 2, 16 with stage 3, and 43 with stage 4 GOLD classification of COPD<br><br>United States and Canada	GOLD stages of COPD	Small airways of lung tissue by GOLD stage of COPD severity	The severity of COPD was strongly associated with increased tissue volume in the wall and greater amount of inflammatory mucous exudates in the lumen of the small airways.	COPD severity is associated with infiltration of the wall by innate and adaptive inflammatory immune cells that make up lymphoid follicles.
Marwick et al., 2004	*In vivo* model that explores the impact of cigarette smoke exposure on inflammation.<br><br>United States and United Kingdom	Cigarette smoke	HDAC activity; histone acetylation levels; phosphoacetylati-on levels;	Cigarette smoke exposure was associated with decreased HDAC2 activity due to protein modification in rat lungs.	There may be a known molecular mechanism by which cigarette smoke exposure influences lung proinflammatory gene transcription and an inflammatory response.
Ito et al., 2005	Study to measure the magnitude of the inflammatory response in the peripheral lung characterized in COPD and test whether this inflammatory response is associated with reduced HDAC activity.<br><br>United Kingdom	Severity of COPD; cigarette smoking;	HDAC and HAT activity in alveolar macrophages and bronchial-biopsy specimens from nonsmokers without COPD, COPD patients with varying GOLD levels, and patients with pneumonia or cystic fibrosis.	As level of COPD severity increased, HDAC activity decreased and IL-8 mRNA and histone-4 acetylation at the IL-8 promoter both increased. mRNA expression of HDAC2, HDAC5, and HDAC8 was reduced in COPD patients with increasing COPD severity grade. Relative to healthy individuals, HDAC activity was reduced and HAT activity remained unchanged in COPD patients' macrophages and biopsy specimens. HAT activity was increased in asthmatic	Patients with COPD gradually decrease total HDAC activity, which is associated with COPD severity.

				patients relative to healthy, non-asthmatic participants.	
Li et al., 2005	Case-control study consisting of 338 children with asthma and 570 matched controls to investigate the association between maternal and grandmaternal smoking timing and childhood asthma<br><br>United States	Maternal and grandmaternal cigarette smoking	Maternal and grandmaternal smoking history; childhood asthma outcome;	Children exposed to prenatal smoking throughout pregnancy had 60% increased odds of asthma development under 5 years of age relative to mothers that didn't smoke during pregnancy. Grandmaternal smoking during pregnancy with the mother was associated with two times the odds asthma development in her grandchildren.	Maternal and grandmaternal smoking *in utero* may increase childhood asthma risk.
Hew et al., 2006	Compare suppression of LPS-induced cytokine release from peripheral blood mononuclear cells of 45 participants consisting of individuals with severe asthma and non-severe asthma and healthy controls<br><br>United Kingdom	Severe, non-severe, and no asthma	Suppression of LPS-induced cytokine release from peripheral blood mononuclear cells	Nuclear HDAC and HAT activity was significantly reduced in participants with severe asthma compared to participants with non-severe asthma.	Asthma may be characterized by decreased HDAC and HAT activity.
White et al., 2006	To determine the methylation status of 6 CpG sites in the proximal promoter of the human IFN-γ in Th1 or Th2 polarized cell lines from children and adult atopics and controls.<br><br>Australia		Methylation levels of 6 CpG sites in the proximal promoter of IFN-γ by bisulphite sequencing	Differentiation of CD4+ T cells through the Th1 pathway showed gradually increasing demethylation of CpG sites in the IFN-γ promoter. Atopy development after 2 years of life wasn't associated with changes in methylation patterns in cord blood T cells.	Differential regulation of IFN-γ promoter methylation in T cells may play a role in atopy development during childhood with further research needed in this area.

Yang et al., 2006	Study to evaluate the molecular mechanisms of inflammatory responses caused by cigarette smoke exposure in human macrophage-like cell line.<br><br>United States	Cigarette smoke	HDAC activity and specific HDAC protein levels; IL-8 and TNF-α release; posttranslational modification of HDACs;	Cigarette smoke exposure increased IL-8 and TNF-α production, relative to the controls, and was associated with increased reactive oxygen species release. Cigarette smoke exposure decreased HDAC activity, and HDAC1, HDAC2, and HDAC3 protein levels.	This study found a vital molecular mechanism within which cigarette smoke influences gene transcription and an inflammatory response in macrophages, which may be useful in future treatment of COPD.
Heijmans et al., 2008	Retrospective cohort study of 971 participants consisting of individuals conceived during the Dutch Hunger Winter, their unexposed same-sex siblings, and healthy controls.<br><br>The Netherlands	Maternal periconception-nal famine nutritional status	Methylation of the *IGF2* differentially methylated regions	individuals who were exposed to *in utero* famine conditions had lower DNA methylation at the *IGF2* gene relative to their unexposed, same-sex siblings	*In utero* nutritional exposures influence DNA methylation of the *IGF2* gene and may lead to increased risk of future NCDs.
Islam et al., 2008	Human fetal lung type II cells<br><br>United States	Glucocorticoid exposure and dexamethasone use	HDAC expression; *SP-A* expression;	Opening of chromatin structure surrounding the surfactant protein-A (*SP-A*) gene in human fetal lung type II cells is associated with decreased expression of HDACs. Glucocorticoids may lead to increased recruitment of HDACs.	Greater knowledge of the pathway and HDAC levels in the presence or absence of glucocorticoids.
Liu et al., 2008	Examine how diesel exhaust particle and allergen exposure changes methylation of IL-4 and IFN-γ promoter genes.<br><br>United States	Diesel exhaust particles and fungus allergen	IgE serum levels; methylation of CpG sites in IL-4 and IFN-γ promoter	Total IgE production increased after allergen exposure and increased further after additional diesel exhaust exposure. This resulted in hypermethylation at CpG245, CpG253, CpG2205 sites of the IFN-γ promoter and	Diesel exhaust and allergen exposures influence methylation of T helper genes adding to evidence of asthma development by environmental exposures.

				hypomethylation at CpG2408 of the IL-4 promoter.	
Baccarelli et al., 2009	1,0997 blood samples from 718 elderly participants<br><br>United States	Traffic particles Ambient particulate pollutants (black carbon, particulate matter [PM2.5], or sulfate)	DNA methylation of (LINE)-1 and Alu repetitive elements by quantitative polymerase chain reaction to estimate the effects on DNA methylation of exposure(s)	Exposure to traffic particles showed decreased repeated-element methylation. LINE-1 methylation decreased after recent exposure to black carbon and traffic particle exposure, was significantly associated with LINE-1 methylation.	Inconclusive on whether decreased methylation leads to exposure-related health effects.
Breton et al., 2009	Bisulfite conversion and pyrosequencing of buccal cells of 348 children and measurement of association between gene-specific CpG methylation differences and smoke exposure in 272 children.<br><br>United States	*In-utero* (prenatal) maternal tobacco smoke	-Methylation of DNA repetitive elements, (LINE)-1 and AluYb8<br>-Gene-specific CpG methylation differences associated with smoke exposure;	DNA methylation patterns were associated with *in-utero* exposure to maternal smoking. Children exposed to prenatal maternal tobacco smoke had significantly lower methylation of AluYb8 and the genes AXL and PTPRO showed significant increases in methylation.	Prenatal tobacco smoke exposure was associated with small and significant alterations in global and CpG-specific DNA methylation, which may alter risks of childhood diseases associated with tobacco smoke.
Izzotti et al., 2009	Measure the lung microRNA expression in rats exposed and unexposed to environmental cigarette smoke<br><br>United States	Environmental cigarette smoke	Expression of microRNAs	Environmental cigarette smoke may alter functions of cell proliferation, apoptosis, differentiation, *Ras* activation, *P53* functions, NF-κB pathway, transforming growth factor–related stress response, and angiogenesis. Various microRNAs were downregulated during environmental cigarette smoke exposure.	Future research is needed to evaluate miRNA profiles of individuals exposed to various environmental hazards and CRD outcomes

Mattes et al., 2009	*In vivo* study of how house dust mite antigens influences development of allergic asthma and its mechanism<br><br>Australia	House dust mites (allergen)	T_H2 response; inflammation; transcription factor activation; GATA3 expression;	Exposure to house dust mite antigens results in start of allergic disease, which is associated with expression of some small, noncoding microRNAs such as miRNA-126.	There is an association between miRNA expression and asthma pathogenesis, which Could be a target for future allergic asthma treatment.
Perera et al., 2009	Cohort study to determine which epigenetic marks were associated with transplacental PAH exposure and/or childhood asthma risk.<br><br>United States	Traffic-related polycyclic aromatic hydrocarbons (PAH)	Prenatal air monitoring for PAH; methylation sensitive restriction fingerprinting in umbilical cord white blood cells DNA	There were 30+ DNA sequences identified in which methylation status was dependent on maternal PAH exposure. Methylation of the ACSL3 59-CGI was associated with maternal airborne PAH exposure over 2.41 ng/m3.	Methylated ACSL3 59CGI in umbilical cord white blood cells may be a marker for transplacental PAH exposure and/or asthma.
Schembri et al., 2009	Study to determine how microRNAs regulate the airway gene expression response to cigarette smoke exposure in bronchial airway epithelium of 20 current and never smokers.<br><br>United States	Cigarette smoke	Whole-genome miRNA and mRNA expression	28 miRNAs were differentially expressed with most miRNAs down-regulated in smokers. Increasing or decreasing mir-218 levels in primary bronchial epithelial cells and H1299 cells resulted in a decrease or increase in the expression of predicted mir-218 mRNA targets, respectively. mir-218 expression decreased in primary bronchial epithelium exposed to cigarette smoke.	mir-218 levels regulate the airway epithelial gene expression response to cigarette smoke exposure and future research is needed to investigate the role for miRNAs in responding to environmental toxins.
Tobi et al., 2009	Cohort of individuals exposed to famine conditions and their same-sex unexposed siblings to evaluate the methylation of 15 loci implicated involved in development and growth.	Prenatal famine nutritional status	Methylation of *INSIGF, IL10, LEP, ABCA1, GNASAS,* and *MEG3*	Methylation of I*NSIGF* was decreased in individuals with prenatal famine exposure relative to unexposed same-sex siblings; while methylation of *IL10, LEP, ABCA1, GNASAS* and *MEG3* increased.	Alterations in DNA methylation may be a result of prenatal famine exposure, which may be dependent on the individual’s sex and time of exposure during

	The Netherlands				gestational development. This may implicate development of CRDs.
Henderson et al., 2010	Subset of a retrospective cohort study including 6,606 children<br><br>United Kingdom	Maternal, prenatal tobacco smoke	Asthma status at age 7.5 years; lung function by spirometry at age 8.5 years; genotyping of maternal and child DNA;	Maternal smoking was associated with reduced childhood FEF25-75 in mother-child pairs with both copies of GSTM1 deleted.	Prenatal tobacco smoke may negatively influence childhood lung function and children's respiratory health may be mediated by other pathways, which need further research.
Koh et al., 2010	Study to investigate the effect of deletion of the complete Th2 local control region on the regulation of Th2 cytokine genes in mice<br><br>South Korea and United States	Allergen	Th2 cytokine expression; histone acetylation levels; DNA methylation in Th2 cytokine loci	The expression of Th2 cytokines significantly decreased in naïve CD4 T cells. Deletion of the local control region resulted in loss of general histone H3 acetylation, decreased histone H3-K4 methylation, and demethylation of DNA in the Th2 cytokine locus.	Th2 local control region's regulation of Th2 cytokine genes plays a vital role in chromatin remodeling of the Th2 cytokine locus and in the pathogenesis of allergic asthma.
Liu, F., et al., 2010<br>*Oncogene*	An *in vitro* study aimed to examine epigenomic effects of cigarette smoke in respiratory epithelia<br><br>United States	Cigarette smoke	Histone methylation levels and expression in human small airway epithelial cells and bronchial epithelial cells	Cigarette smoke exposure may lead to gradually increasing genomic hypomethylation and locoregional DNA hypermethylation. Cigarette smoke exposure showed 'cancer-associated' epigenomic alterations in respiratory epithelia samples.	This study on cigarette smoke exposure requires further research on early epigenetic mechanisms regulating gene expression in the development of lung cancer.
Liu, G., et al., 2010<br>*The Journal of Experimental Medicine*	To explore the role miRNA, specifically miR-21 in fibrotic lung diseases<br><br>United States	Uncontrolled extracellular matrix production by fibroblasts	miRNA regulation and expression;	There was up-regulation of miR-21 in mice lungs with IPF.	miR-21 may play a vital role in IPF and fibrotic lung diseases, suggesting a potential therapeutic target for treatment.

Pandit et al., 2010	RNA from 10 control and 10 IPF tissues to determine changes in expression and the role of microRNAs in IPF.<br><br>United States		Expression of let-7d measured by real-time PCR and *in situ* hybridization	In lung epithelial cells, let-7d is a microRNA down-regulated in IPF and HMGA2 is overexpressed.	Down-regulation of let-7 microRNAs<br>may be a vital marker to recognize the lung phenotype in IPF.
Suter et al., 2010	Cohort of smokers and healthy control participants assessing placental expression of multiple CYP family<br><br>United States	*In-utero* maternal cigarette smoke	*CYP1A1* placental expression; DNA methylation; sequencing of the entire proximal 1-kilobase promoter;	*In-utero* tobacco exposure increases placental *CYP1A1* expression in association with differential methylation at a critical response element.	There is an epigenetic correlation between increased expression of placental *CYP1A1* and Resulting DNA adduct accumulation with future research needed to study the epigenomic-environmental interactions between *in-utero* maternal cigarette smoke and CRDs.
Waterland et al., 2010	Study to identify genomic regions that show interindividual epigenetic variation and understand how DNA methylation at putative human metastable epialleles is influenced by maternal environment at conception.<br><br>The Gambia and Malawi	Maternal nutrition status at conception and environment	Two-tissue parallel epigenomic screening method in putative metastable epialleles in the human genome	DNA methylation at metastable epialleles increased in participants conceived during rainy season (lower nutritional status compared to dry season).	These differences in DNA methylation in metastable epialleles may explain inter-individual epigenetic variation that may be implicated in CRDs.
Ezzie et al., 2012	Lung tissue of 35 participants consisting of 9 and 26 smokers without COPD and with COPD, respectively.	Tobacco smoke	miRNA and mRNA expression	70 miRNAs such as miR-223 and miR-1274a and 2667 mRNAs were differentially expressed between lung tissue from smoking participants with and without COPD. miR-15b	miRNA and mRNA are differentially expressed in individuals with COPD compared without COPD.

	United States			was increased in COPD participants compared to participants without COPD, and differentially expressed based on COPD severity.	
Fu et al., 2012	Prospective cohort study with analysis consisting of 60 children with mild asthma and 122 children with severe asthma<br><br>United States	Indoor NO_2 exposure (asthmogen) and *ADRB2* methylation	*ADRB2* 5'-UTR methylation levels in blood samples and effects between NO_2 exposure and *ADRB2* 5'-UTR methylation using methylation-specific PCR.	There was a significant association between intermediate and high levels of *ADRB2* methylation and severe childhood asthma. There was 4.5 times greater odds of severe asthma among children exposed to indoor NO_2 exhibiting high *ADRB2* methylation levels.	*ADRB2* 5'-UTR methylation may mark risk of asthma severity and NO_2-associated asthma exacerbations in children and a possible epigenetic link between NO_2 and childhood asthma severity.
Kohli et al., 2012	Study to evaluate the association between secondhand smoke and ambient air pollution with DNA methylation and expression in 102 children from cities of high and low ambient air pollution levels exposed and unexposed to secondhand smoke.<br><br>United States	Secondhand smoke; ambient air pollution	DNA methylation and expression of IFN-γ and Foxp3 in T cells	Secondhand smoke and ambient air pollution exposures were associated with hypermethylation and decreased expression of IFN-γ in T effector cells and Foxp3 in T regulatory cells.	This mechanism in which secondhand smoke and ambient air pollution may be associated with development and progression of asthma and allergies with further research needed.
Morales et al., 2012	Cohort study of 358 children to identify DNA methylation patterns of asthma-related phenotypes in childhood, and to understand the role of prenatal exposures on these epigenetic marks	Maternal smoking; folate supplementati-on	DNA methylation; odds of outcome (asthma)	DNA methylation at a CpG site in the *ALOX12* gene was decreased in children having persistent wheezing relative to those that never wheezed. DNA hypomethylation at ALOX12 loci was associated with higher risk of persistent wheezing.	DNA methylation of *ALOX12* may be an epigenetic biomarker for asthma.

	Spain				
Qiu et al., 2012	Two family-based cohort studies consisting of 1,085 and 369 participants with nonneoplastic lung disease to assess methylation marks in their DNA.<br><br>United States	Cigarette smoke	DNA methylation marks using array-based methylation screens	There were 349 CpG sites associated with the presence and severity of COPD. The hypomethylation of *SERPINA1* was associated with COPD and lower lung function.	Epigenetic pathways may both contribute to COPD with DNA methylation as a promising biomarker for COPD development for future study.
Rabinovich et al., 2012	Immunoprecipitated methylated DNA from 12 IPF lungs, 10 lung adenocarcinomas and 10 healthy lungs<br><br>United States and Israel		BRB-Array Tools	The expression of genes STK17B, STK3 and HIST1H2AH with hypomethylated promoters was increased in IPF lungs. IPF lung methylation profile was similar to lung cancer and partly similar to control lungs. IPF lungs did not exhibit hypomethylation of LINE-1 retrotransposon even though lung cancer lungs did.	The similarity in epigenetic marks between IPF and cancer may constitute similar pathogenetic mechanisms. Further research is needed to understand these specific differences.
Runyon et al., 2012	Cohort study of 21 monozygotic twins discordant for asthma with the aim of determining whether epigenetic modifications in T cells were associated with asthma and second hand smoke exposures.<br><br>United States	Secondhand smoke	Regulatory T cell and effector T cell level of function, protein expression; CpG methylation; current asthma status; secondhand smoke exposure	Regulatory T cells of asthmatic discordant twins showed decreased FOXP3 protein expression, which was associated with increased levels of CpG methylation in the FOXP3 locus relative to other non-asthmatic twin. Secondhand smoke exposure was associated with modifications in both regulatory and effector T cells at the transcriptional level in asthmatics.	Secondhand smoke exposure may alter T cell responses associated with asthma.

Brunst et al., 2013	Cohort study of 92 children with DNA from saliva samples and asthma symptom history and diagnosis<br><br>United States	Diesel exhaust particles exposure without exposure to environmental tobacco smoke	*FOXP3* methylation, diesel exhaust particle exposure, asthma development, history of wheezing	Increased FOXP3 methylation was associated with increased odds of experiencing persistent wheezing and early transient wheezing during childhood compared to children that hadn't wheezed. Children with increased FOXP3 methylation had 112% increased odds of developing asthma relative to children with lower FOXP3 methylation	FOXP3 methylation is associated with repeated childhood diesel exhaust particle exposure and increased risk for developing persistent wheezing and asthma.
Lam et al., 2013	Explore the mechanisms that autophagy regulates airway function and ciliated epithelial cell length when exposed to cigarette smoke in mice tracheal epithelial cells and human COPD specimens<br><br>United States	Cigarette smoke	Cilia length; autophagic turnover of ciliary proteins;	Cigarette smoke exposure showed decreased cilia length and induced autophagy in mice tracheal epithelial cells. Human specimens with COPD showed epigenetic deregulation of HDAC6 by hypomethylation and increased protein expression in the airways.	An autophagy-dependent pathway regulates cilia length in the presence of cigarette smoke exposure which with further research could be a therapeutic avenue for COPD.
Moreno-Macías et al., 2013	Two cohort studies of 257 asthmatic children to investigate the relationship between dietary intake of vitamin C, high-ozone exposure, and lung function.<br><br>Mexico	Ozone exposure; vitamin C intake;	Baseline diet; baseline blood samples; FEF by spirometry; ozone exposure	Persistent asthmatic children with 4-6 risk alleles and classified as low vitamin C intake had an significant average reduction in FEF_{25-75} of 97.2 ml/s per 60 ppb of ozone. However, in children with 1-3 risk alleles the effect of ozone on lung function did not change by vitamin C intake.	Dietary vitamin C intake may be protective against the effect of ozone exposure on lung function in asthmatic children living in areas of high ozone exposure.
Dominguez-Salas et al., 2014	Prospective study to measure the influence of mothers' peri-conceptional dietary intakes and	Maternal Diet	DNA methylation in metastable epialleles of Gambian women	Maternal nutritional status during early pregnancy can lead to persistent and systemic epigenetic changes in	Maternal peri-conceptional nutritional status can influence the child's DNA methylation

	plasma concentrations<br><br>The Gambia		during rainy and dry seasons	human metastable epialleles	level and epigenome, potentially leading to future and lifelong CRDs.
Jiang et al., 2014	Double-blind crossover study of filtered air and diesel exhaust exposures with 16 non-smoking asthmatics<br><br>Canada	Diesel exhaust exposure (short-term)	Pre-exposure and post-exposure (6 and 30 hrs) blood samples measuring mononuclear cell DNA methylation	DNA methylation at 2827 CpG sites were altered by diesel exhaust exposure relative to filtered air.<br>CpG sites mainly decreased in methylation after diesel exhaust exposure, especially at a site residing within *GSTP1* and CpG sites overlapping with Alu and LINE1 elements.	Short-term exposure to diesel exhaust resulted in DNA methylation changes at CpG sites in genes with inflammation and oxidative stress response functions and microRNA. Diesel exhaust exposure's role in DNA methylation may be implicated in CRD development.
Joubert et al., 2014	Cohort study of maternal plasma and cord blood from 1,068 births<br><br>Norway	Maternal and maternal grandmother cigarette smoking	DNA methylation in cord blood at 26 previously identified CpGs	*In utero* cigarette smoke exposure showed DNA methylation at 26 loci of newborns when the mother smoked past 18 weeks gestation rather than quitting earlier in pregnancy.	Differential methylation at these CpGs in children as a result of cigarette smoke may lead to long lasting effects on methylation.
Markunas et al., 2014	Epigenome-wide association study with the aim of exploring changes in DNA methylation in infants as a result of prenatal maternal tobacco smoking<br><br>Norway	*In utero* maternal tobacco smoking	DNA methylation in whole blood in infants; maternal tobacco smoking status;	There were 185 CpGs with altered methylation status in infants of smokers including gene regions such as *FRMD4A, ATP9A, GALNT2*, and *MEG3* (involved in embryonic development among other functions).	Further research is needed to explore how altered methylation of embryonic development gene regions influences the later health outcomes associated with *in utero* maternal smoking exposure.
Vucic et al., 2014	Matched case-control study to determine whether DNA methylation patterns are altered in small airway epithelia of former smokers with and without COPD.	Cigarette smoke	Genome-wide methylation and gene expression analysis in small airway epithelial	DNA methylation is genome-wide in small airways of COPD patients and associated with changes in expression of genes and pathways vital to COPD (NF-E2–related factor 2	This research characterizes potential COPD epigenetic markers and targets of COPD treatment interventions.

	Canada		DNA and RNA collected during bronchoscopy	oxidative response pathway).	
Hernandez-Vargas et al., 2015	Cohort study of 115 pregnant women evaluating DNA methylation status of white blood cells from children 2-8 months old<br><br>The Gambia	Maternal aflatoxin (a liver carcinogen and contaminant of staple foods in sub-Saharan Africa) exposure	DNA methylation status of white blood cells; genome-wide DNA methylation of infant white blood cells; aflatoxin exposure in mothers measured using ELISA to measure albumin adducts in plasma	Prenatal aflatoxin exposure was significantly associated with DNA methylation in their children at 71 CpG sites. Aflatoxin-associated differential methylation was found in the genes FGF12, IGF1, CCL28, TLR2, and TGFBI.	Prenatal aflatoxin exposure to aflatoxin was associated with differential DNA methylation patterns in genes involved in immune function with the relationship to CRD development needing further research.
Murphy et al., 2015	Cohort study to explore the association between methylomic variation in early childhood and asthma symptoms in 37 monozygotic twin pairs.<br><br>United Kingdom	Variety of environmental factors	DNA methylation patterns in buccal cell samples	DNA methylation at individual CpG sites were variable within discordant twin pairs especially at the differentially methylated position located in the *HGSNAT* gene.	There were DNA methylation differences associated with childhood asthma that lasted into early adulthood.
Yang et al., 2015	To evaluate the association between epigenetic changes in circulating peripheral blood mononuclear cells and allergic asthma in inner-city atopic asthmatic children and healthy controls.<br><br>United States	Urban environment	DNA methylation patterns and gene expression in from DNA and RNA in peripheral blood mononuclear cells from study participants.	There were 81 differentially methylated regions with several (*IL13, RUNX3*, and *TIGIT*) hypomethylated in asthmatics. Hypomethylated and hypermethylated regions were significantly associated with increased (for asthma) and decreased (for IgE) gene expression.	Specific DNA methylation marks in various gene loci are associated with asthma highlighting epigenetic changes that may be involved in asthma development.

Somineni et al., 2016	Study to explore the associations between *TET1* methylation and asthma and traffic-related air pollution in 12 asthmatic African-American children and their non-asthmatic siblings.<br><br>United States	Traffic-related air pollution	*TET1* methylation levels from DNA from nasal airway epithelial cells; traffic-related air pollution exposure; methylation studies in saliva, peripheral blood mononuclear cells and human bronchial epithelial cells;	Asthma was associated with loss of methylation at a single CpG site in the *TET1* promoter (cg23602092) and increased global 5hmC. Traffic-related air pollution exposure was associated with increased methylation at the same site.	*TET1* DNA methylation may be a viable marker for asthma and in individuals exposed to traffic-related air pollution.
Clifford et al., 2017	Randomized crossover-controlled exposure study in human bronchial epithelial cells<br><br>Canada	Allergen and diesel exhaust particle exposure	Single-site (CpG) resolution global DNA methylation	Allergen, diesel exhaust alone, or both allergen and diesel exhaust exposures showed significant changes in 7 CpG sites after 2 days. When allergen and diesel exhaust exposures were separated by 4 weeks there were significant changes in more than 500 sites.	The order and time between allergen and diesel exhaust particle exposure can prime the lung for alterations in DNA methylation and future development of CRDs

Abbreviations: ALOX12: arachidonate 12-lipoxygenase; COPD: chronic obstructive pulmonary disease; CRD: chronic respiratory disease; ELISA: enzyme-linked immunosorbent assay; Foxp3: forkhead box protein 3; GOLD: Global Initiative for Chronic Obstructive Lung Disease; HAT: histone acetylases; HDAC: histone deacetylases; IFN-γ: interferon-gamma; IPF: idiopathic pulmonary fibrosis; LINE-1: long interspersed nucleotide element; NCDs: non-communicable diseases; PCR: Polymerase Chain Reaction; TNF- α: tumor necrosis factor alpha;
